# Vascular endothelial primary cilia regulate blood flow-induced EndoMT

**DOI:** 10.1186/2046-2530-1-S1-P70

**Published:** 2012-11-16

**Authors:** B Hierck, AD Egorova, RE Poelmann

**Affiliations:** 1 Leiden University Medical Center, the Netherlands

## 

Vascular endothelial cells present cilia in response to low and oscillatory flow. Cilia function in transducing local blood flow information into functional responses, like nitric oxide production and initiation of gene expression. In the embryonic heart high flow regime applies to the endocardial cushion area, and the absence of cilia here coincides with the process of endothelial-to-mesenchymal transition (EndoMT). During this transdifferentiation process EC lose their endothelial characteristics, gain a mesenchymal phenotype, and migrate into the cardiac jelly to form the primordia of the cardiac valves. In this study we investigate the role of the primary cilium in further defining the responses of EC to fluid shear stress and in EndoMT. We used embryonic EC from the *IFT88Tg737RPW* (or *Tg737orpk/orpk*) mouse, and compared these to ciliated EC under dynamic flow conditions. *In vitro*, non-ciliated *Tg737orpk/orpk* EC undergo flow-induced EndoMT which is dependent on downregulation of the transcription factor Klf4. Ciliated wild type (WT) cells retain an epithelial phenotype under these conditions. However, when exposed to higher shear levels WT cells lose their cilia and undergo EndoMT, gaining a phenotype closely resembling that of *Tg737orpk/orpk* EC under flow. This Tgfβ/Alk5 dependent transformation is prevented by blocking Tgfβ signaling, overexpression of Klf4, or rescue of the primary cilium. This study demonstrates the central role of primary cilia in rendering EC prone to shear-induced activation of Tgfβ/Alk5 signaling and EndoMT, and thereby provides a functional link between primary cilia and flow related endothelial performance.

